# The effect of COVID‐19 on surgical management of skin cancers of the head, face and neck in elderly patients

**DOI:** 10.1002/ski2.175

**Published:** 2023-03-28

**Authors:** Tiffanie‐Marie Borg, Jessica Eisold, Yusuf Miyanjo, Elena Pappa

**Affiliations:** ^1^ Department of Plastic and Reconstructive Surgery Queen's Hospital London UK; ^2^ Department of Dermatology Queen's Hospital London UK; ^3^ Department of Oral and Maxillofacial Surgery Queen's Hospital London UK

## Abstract

**Background:**

The Corona Virus (COVID‐19) has had a profound impact on healthcare systems worldwide, with interruptions to medical practices including the delivery of cancer treatment. Skin cancer is one of the leading causes of malignancy worldwide, with later stages of disease correlating to poorer prognosis. Immunocompromized and elderly patients represent populations that are at higher risk for adverse outcomes related to skin cancer, treatment delay and COVID‐19 infection.

**Methodology:**

Patients aged 65 and above who underwent surgical management of skin cancers from 31 January 2020 to 31 January 2021 were included in this study then compared with samples pre‐ and post‐pandemic. Retrospective analysis was performed regarding: date of referral to date of surgery, skin cancer type, location of cancer, surgery performed, anaesthesia used, sutures used and outcomes. Data was compared to national guidelines.

**Results:**

Five hundred and twenty skin cancers were included in this analysis, of which 340 were treated during the COVID‐19 pandemic. Of the cohort treated during the pandemic, 44.2% (*n* = 111) received excision and direct closure, 13.1% (*n* = 33) underwent reconstruction by integra dermal substitute, 3.2% (*n* = 8) by split thickness skin graft, 6.4% (*n* = 16) by full thickness skin grafts and 33.1% (*n* = 83) by local flaps. Complete excision was achieved in 88.5% of cases (*n* = 301). The mean time from referral to surgery was 119 days. There were no deaths associated with COVID‐19.

**Conclusion:**

Safe and prompt treatment of head and neck skin cancers is achievable despite the COVID‐19 pandemic. Measures to minimize infection risk include the use of teledermatology, reliable COVID‐19 testing, Green Pathways and a reduction in the mean referral to surgery time.

1

**What is already known about this topic?**

- Skin cancer represents the most common malignancy worldwide. The UK Healthcare system was profoundly affected by the corona virus (COVID‐19). Cancer diagnostics and surgery were disrupted by the response of the National Health Service to the pandemic. Progression of cancers during delay will impact patient's survival. Hanna et al (2020) report a 6%–8% increased risk of death for head and neck cancers with every 4 week delay in treatment. In this context the aim of cancer management includes balancing between the risk of cancer progression and that of infection. Management guidelines for skin cancer care during the COVID‐19 pandemic have been released.

**What does this study add?**

- There is a need for skin cancer guidance in relation to COVID‐19 that considers elderly patients who are at higher risk of complications from COVID‐19 infection.
- This study presents the impact of COVID‐19 on the surgical management of skin cancers of the head and neck in elderly patients. To continue to improve upon current practices this study identified ways to provide skin cancer care while minimizing the risk of COVID‐19 infection.

## 2 INTRODUCTION

Skin cancers are one of the leading causes of malignancy worldwide.^1^ One in three malignancies reported are skin cancer, representing the most common neoplasm.^2^ They are a heterogeneous group of cancers comprizing melanoma and non‐melanoma skin cancer (NMSC). With increased sun exposure and an ageing population,^3^ the incidence rates of skin cancer are rising, posing a public health threat globally.^4^ ^,^ ^5^

The COVID‐19 pandemic has had a profound impact on the healthcare system in the United Kingdom (UK), resulting in widespread disruptions to medical practices, including the delivery of routine dermatologic care.^6^ ^,^ ^7^ As a result, patients have experienced delayed treatment and underestimation of malignant changes.^8^ From April 2020 to November 2020 there was a 28% decrease in the diagnosis of melanoma, and a 24% decrease in diagnoses of all skin cancers (excluding NMSC).^9^ The treatment of advanced skin cancers correlates with a poorer prognosis and significant healthcare burden.^10^ For every 4‐week delay in treatment for head and neck cancers, there is a 6%–8% increase in the risk of death.^11^ This highlights the importance of prompt treatment while balancing the risks of COVID‐19 infection.

With the arrival of COVID‐19 in the UK in January 2020, the British Association of Dermatology updated its guidelines,^12^ with advice including considering (i) cancelling elective surgeries, (ii) deferring surgical excisions of basal cell carcinomas for 3–6 months, (iii) deferring excisions of in situ and small, well differentiated squamous cell carcinomas and (iv) opting for definitive surgery over excisional biopsies. Highly symptomatic lesions with the potential for rapid growth should continue to be prioritized for surgical intervention. Though COVID‐19 is no longer at its greatest peak, infections continue to pose a risk to patients admitted to hospitals. It is essential that skin cancers, including elective cases, are treated in a timely manner while minimizing infection risk of hospital acquired infections during admissions.

Skin cancer has a higher prevalence in elderly patients.^13^ Skin ageing involves changes to both the dermal and epidermal layers rendering increased susceptibility to skin cancers. Immunocompromized individuals are more susceptible to nosocomial infections. This places them at an increased risk of COVID‐19 infection and the complications associated with it.^14^ Diagnostic delays disproportionately impact the elderly population.^15^ There is therefore a need to administer skin cancer services in a safe manner for elderly and immunocompromized patients, despite the continued effects of COVID‐19.

## 3 METHODOLOGY

This study aims to assess the impact of COVID‐19 on the surgical management of skin cancers of the head and neck in elderly patients at Barking, Havering and Redbridge University Hospitals NHS Trust in London, United Kingdom. Operative skin cancer treatment was performed by two consultant senior surgeons in line with current recommended practice.

Elderly patients (aged 65 years and above) with confirmed diagnosis of skin cancer of the head and/or neck were included in this analysis. Patients who underwent biopsies for suspected cancer who were found to have non‐cancerous lesions on pathology were excluded.

The focus of patients included in this study includes those treated based on the arrival of COVID‐19 in the United Kingdom; the first COVID‐19 case was on 29 January 2020.^16^ Patients who underwent surgical management from 31 January 2020 to 31 January 2021 were retrospectively reviewed according to: (i) date of referral to date of surgery, (ii) histological type of cancer and location, (iii) type of reconstruction performed, (iv) anaesthesia used, (v) suture types used and (vi) outcome. Findings were then compared to national guidelines. Areas of good practice as well as areas for improvement were identified to optimize patient care. These findings were then compared to a cohort of patients treated prior to the pandemic (between the 1 January 2018 and 20 May 2018), as well as after a cohort of patients treated later between 1 December 2021 and 1 March 2022, when hospital staff were more accustomed to working despite the presence of COVID‐19.

## 4 RESULTS

Five hundred and twenty skin cancers, including basal cell carcinoma, squamous cell carcinoma, malignant melanoma and pleomorphic sarcoma were treated in the timeframe of this analysis (Table 1). Of these, 340 patients were treated in the year of the pandemic while a comparative cohort of 50 and 130 patients were reviewed pre‐ and post‐prime pandemic period. Regarding the location, the majority of skin cancers treated during the pandemic were located on the scalp (*n* = 64), nose (*n* = 54) or ear (*n* = 47). The most common site of cancer treated in the pre‐pandemic cohort was the nose, while the cheek was most treated in the post‐pandemic cohort. The full breakdown of cancer site is summarized in Table 2. The mean time to surgery from referral was 119 days during the pandemic. This is an improvement to treatment times pre‐pandemic and has continued to improve when compared to latter data obtained from 1 December 2021 to 1 April 2022 (Table 3).

**TABLE 1:** Skin cancers included in this analysis (*n* = 520)

Skin cancer	1 January 2018–20 May 2018		31 January 2020–31 January 2021		1 December 2021–1 April 2022	
	*n*	%	*n*	%	*n*	%
Basal cell carcinoma	32	64	182	53.5	70	53.8
Squamous cell carcinoma	17	34	145	42.6	54	41.5
Malignant melanoma	1	2	12	0.04	6	4.6
Pleomorphic sarcoma	0	0	1	0.003	0	0
Total	*n* = 50		*n* = 340		*n* = 130	

**TABLE 2:** Location of skin cancers included in this analysis (*n* = 520)

Cancer site	1 January 2018–20 May 2018		31 January 2020–31 January 2021		1 December 2021–1 April 2022	
	*n*	%	*n*	%	*n*	%
Scalp	9	18	64	18.8	24	18.4
Temple	4	8	40	11.8	14	10.7
Ear	6	12	47	13.8	15	11.5
Preauricular region	4	8	8	2.4	4	3.1
Postauricular region	2	4	4	1.2	0	0
Forehead	3	6	40	11.8	15	11.5
Periorbital region	1	2	12	3.5	3	2.3
Nose	11	22	54	15.9	20	15.4
Cheek	4	8	45	13.2	25	19.2
Lip: Skin above vermilion border	5	10	14	4.1	1	0.8
Chin	0	0	4	1.2	2	1.5
Neck	1	2	8	2.4	7	5.4

**TABLE 3:** Mean time to surgery from referral

Skin cancer	Mean time to surgery (days)		
	1 January 2018–20 May 2018	31 January 2020–31 January /2021	1 December 2021–1 April 2022
Basal cell carcinoma	170.2	139.6	136
Squamous cell carcinoma	87.7	60.6	48
Malignant melanoma	19	60.7	32
Pleomorphic sarcoma	N/A	64	N/A
Overall mean	139.2	119	94.7

Electronic data regarding surgical reconstruction was available for 372 skin cancers. The operative notes for the remaining cases were hand‐written. Due to COVID‐19 restrictions these were not available. Pre‐pandemic, local flaps were the most commonly used reconstructive technique post‐excision, accounting for 52% of cases. This reduced during the pandemic where 83 (33.1%) cancers received local flap reconstruction while 111 (44.2%) were closed directly. During the pandemic, integra dermal substitute was used for 33 cancers while remaining cancers were covered using full (*n* = 16) or split (*n* = 8) thickness skin grafts. In the patient cohort treated 1 December 2021–1 April 2022, the proportion of patients receiving local flaps increased compared with the pandemic cohort but the majority (43.7%) received direct skin closure post cancer excision. The reconstruction used following skin cancer excision is demonstrated in Table 4.

**TABLE 4:** Reconstruction of skin cancers following excision

Cancer site	1 January 2018–20 May 2018		31 January 2020–31 January 2021		1 December 2021–1 April 2022	
	*n*	%	*n*	%	*n*	%
Direct skin closure	19	38	111	44.2	31	43.7
Integra dermal substitute	0	0	33	13.1	9	12.7
Split thickness skin graft	4	8	8	3.2	2	2.8
Full thickness skin graft	1	2	16	6.4	1	1.4
Local flap	26	52	83	33.1	28	39.4
Advancement flap	(11)		(55)		(11)	
Rotation flap	(1)		(12)		(7)	
Transposition flap	(2)		(16)		(2)	
Not specified	(12)		(0)		(8)	
**Total**	*n* = 50		*n* = 251		*n* = 71	

Sutures used were dissolvable in all cases (4.0–6.0 Monocryl or Vicryl Rapide). During the pandemic, a higher proportion of patients were treated with local anaesthesia. In this cohort, lignocaine with adrenaline was used for 97% cases (*n* = 330). Analgesia used in each cohort is demonstrated in Table 5.

**TABLE 5:** Anaesthetic used for patients treated in this analysis

Anaesthetic	1 January 2018–20 May 2018		31 January 2020–31 January 2021		1 December 2021–1 April 2022	
	*n*	%	*n*	%	*n*	%
Local	43	86	330	97	124	95
General	7	14	10	3	6	5

During the pandemic, complete excision was achieved in 90.2% of cases (*n* = 307). Twenty one incomplete excisions were basal cell carcinomas while 12 cases were squamous cell carcinomas. No patients died from COVID‐19 following their surgical skin cancer treatment. In the pre‐ and post‐pandemic cohort where in each cohort there was one incomplete basal cell carcinoma excision that was re‐operated without complication (Table 6). This analysis shows safe delivery of skin cancer care, despite COVID‐19.

**TABLE 6:** Patient outcomes before, during and after the pandemic

Anaesthetic	1 January 2018–20 May 2018		31 January 2020–31 January 2021		1 December 2021–1 April 2022	
	*n*	%	*n*	%	*n*	%
Complete excision, discharged	25	50	182	53.5	19	47.5
Complete excision, follow‐up continued	23	46	125	36.7	17	42.5
For further radiotherapy	1	2	0	0	3	7.5
Incomplete excision, reoperated	1	2	33	9.7	1	2.5

## 5 DISCUSSION

Non‐melanoma skin cancer is the most common cancer in fair‐skinned individuals.^17^ In white populations, cutaneous melanoma is the most rapidly rising malignancy, with a five‐fold increase in incidence over the past 30 years.^17^ The increasing incidence is partly due to increased UV radiation exposure, with most skin cancers occurring in sun‐exposed sites. In our analysis 96.5% of cancers were non‐melanoma. The highest proportion of skin cancers treated during the pandemic were on the scalp (18.8%), nose (15.9%) and ear (13.8%). 44.2% (*n* = 111) of patients underwent skin cancer excision and direct skin closure while 33.1% (*n* = 83) received local flap reconstruction. Compared with data collected for patients treated pre‐pandemic this demonstrates a shift from treatment by local flaps to direct closure. During the pandemic, Integra dermal substitute was used for 13.1% of patients (*n* = 33) whereas split thickness and full thickness skin grafts were used for 3.2% (*n* = 8) and 6.4% (*n* = 16) of patients respectively. In this patient cohort Integra demonstrated good epithelialization. These patients healed well without requiring re‐attendance to the hospital for further skin grafting procedures or dressing changes during the pandemic, serving as a good reconstructive option for small/medium‐sized skin cancers.

In response to the pandemic, the British association of Dermatology suggested cancelling elective procedures and limiting emergency procedures, thereby prioritizing squamous cell carcinomas and malignant melanomas. However, COVID‐19 has had prolonged effects on healthcare systems. Delaying skin cancer treatment increases patient risk of developing further complications, the need for more complex surgery,^18^ and the mortality rates.^19^ This study highlights the negative impact of COVID‐19 on the access for timely intervention of skin cancers. Nationally the target for initial NMSC treatment is within 6 weeks of referral from Dermatology or General Practice.^20^ To achieve this, patients are referred on a 2‐week‐wait referral basis. Local recommendations advocate NMSC treatment within 31 days, with melanoma treatment ideally within 28 days. In this analysis the average time for basal cell carcinomas was 139.6 days, squamous cell carcinomas 60.6 days and melanomas 60.7 days.

Local protocols were implemented throughout the United Kingdom to streamline the process of preparing patients for surgery and minimizing risk for both patients and staff.^21^ In our unit, teledermatology proved to be an extremely effective option, enabling joined clinical assessment, as well as virtual medical photography of patients pre‐ and post‐operatively. This enabled continuation of care, allowing patient cases to be prioritized while limiting patient exposure to the virus.^22^ Post‐operatively, this also ensured patients were healing well without risking health by attending the hospital. The use of dissolvable sutures in all patients reduced patient visits to the hospital or general practitioner for removal in dressing clinics. Where patients were required to attend the hospital, risk of infection was minimized by limiting appointments with joint clinics with the Dermatology and Maxillofacial teams.

Of the 340 cases treated during the pandemic, there were 33 incomplete excisions (21 basal cell and 12 squamous cell carcinomas). In all incompletely excised basal cell carcinomas, the pathology report noted an infiltrative pattern of growth with incomplete excision at the periphery of the excision in 10 cases and focally incomplete excision in two cases. Incomplete squamous cell carcinoma excisions included clear peripheral margins. However, the lesions had deep infiltration down to bone and/or cartilage. All incompletely excised basal cell carcinomas were re‐excised later. Squamous cell carcinomas were followed up, discussed by the multi‐disciplinary team then further treated on a case‐by‐case basis. Patients with incomplete excisions received Mohs micrographic surgery treatment by liaising with Guy's and St Thomas' Hospital in London, following which reconstruction was performed locally. Radiotherapy for first stage skin cancers was stopped during this study period, in line with national recommendations.

Of the 340 skin cancer cases treated during the pandemic, there were no COVID‐19 related deaths. These positive outcomes are likely due to various measures that were taken during the pandemic to minimize patient exposure to the virus. Early in the pandemic, operating theatres in the private sector were used to minimize patient exposure to COVID‐19. King George Hospital later became a ‘Green‐zone’ hospital hub, whereby all patients and staff had a negative COVID‐19 swab prior to arrival at the hospital. Meanwhile, Queen's Hospital remained an ‘orange/red’ hospital that accepted patients requiring emergency non‐skin cancer surgeries, regardless of their COVID‐19 status. Over the course of the pandemic, there was a transition from requiring operating theatres in the private sector to making use of the ‘Green theatres’ at King George Hospital. Later, skin cancer lists were re‐introduced to Queen's Hospital. To minimize infection risk here, skin cancer theatres were separated as ‘Green‐only’ zones. The use of this ‘green pathway’ is particularly important to minimize COVID‐19 transmission in the elderly, especially if immunocompromized.

During the pandemic the number of patients treated under general anaesthetic significantly reduced throughout the United Kingdom,^23^ and as demonstrated by this data highlighting a fall from 14% to 3% of patients receiving general anaesthesia. This is likely due to the increased risk of viral spread during intubation, as well as resulting inpatient hospital stay. In this analysis, the majority of small skin cancers were successfully treated using local anaesthesia regardless of the pandemic due to their multiple comorbidities, in whom general anaesthesia poses a greater risk. Minor skin cancers treatable with local anaesthesia may be excised in outpatient clinics, freeing main theatres.

## 6 CONCLUSION

To continue to improve upon current practices this study identified ways to minimize hospital appointments and decrease time to surgery. Teledermatology should be utilized to its fullest extent, including patient reviews and medical photography. Where patients need to attend the hospital, this should be done after testing negatively for COVID‐19. Minor operations can take place in outpatient clinics that have been properly equipped with surgical instruments. Surgical theatres are to continue utilizing the Green Pathway with patients remaining in Green areas of the hospital. At present, the mean time from referral to surgery is 119 days. We recommend that surgery should be completed within 31 days of referral, which is half the time recommended by national guidelines.

## CONFLICT OF INTEREST

None to declare.

## AUTHOR CONTRIBUTIONS

**Tiffanie‐Marie Borg**: Conceptualization (Equal); Data curation (Equal); Formal analysis (Equal); Investigation (Equal); Methodology (Equal); Project administration (Equal); Supervision (Equal); Validation (Equal); Visualization (Equal); Writing – original draft (Equal); Writing – review & editing (Equal). **Jessica Eisold**: Data curation (Equal); Formal analysis (Equal); Investigation (Equal); Methodology (Equal); Writing – original draft (Equal); Writing – review & editing (Equal). **Yusuf Miyanji**: Data curation (Equal); Formal analysis (Equal); Investigation (Equal); Methodology (Equal); Writing – original draft (Equal); Writing – review & editing (Equal). **Elena Pappa**: Conceptualization (Equal); Supervision (Equal); Visualization (Equal); Writing – review & editing (Equal).

## ETHICS STATEMENT

Ethical approval was not required for this retrospective analysis. However, all procedures performed were in accordance with the ethical standards of the institutional and/or national research committee and with the 1964 Helsinki declaration and its later amendments or comparable ethical standards.

## Data Availability

The data that support the findings of this study are available from the corresponding author upon reasonable request.
